# Editorial: Therapeutic relevance and mechanisms of neuro-immune communication in brain injury

**DOI:** 10.3389/fncel.2023.1209083

**Published:** 2023-08-01

**Authors:** Pengyue Zhang, Yulong Bai, Feng Zhang, Xiangjian Zhang, Yunping Deng, Yuchuan Ding

**Affiliations:** ^1^Institute of Acupuncture, Tuina and Rehabilitation, The Second Clinical Medical School, Yunnan University of Traditional Chinese Medicine, Kunming, China; ^2^Department of Rehabilitation Medicine, Huashan Hospital Affiliated to Fudan University, Shanghai, China; ^3^Department of Rehabilitation Medicine, The Third Hospital of Hebei Medical University, Shijiazhuang, China; ^4^Department of Neurology, Second Hospital of Hebei Medical University, Shijiazhuang, China; ^5^Department of Anatomy and Neurobiology, University of Tennessee Health Science Center (UTHSC), Memphis, TN, United States; ^6^Department of Neurosurgery, Wayne State University School of Medicine, Detroit, MI, United States

**Keywords:** neuro-immune communication, inflammatory, brain injury, therapeutic strategy, neuroprotection

For many years, the central nervous system (CNS) was thought to be immune-privileged due to the presence of the blood-brain barrier (BBB) and the absence of lymphatic vasculature. However, recent studies have uncovered the intricate interactions between the CNS and the immune system. In particular, microglia–resident immune cells in the brain–share developmental origins and functions with monocytes in the immune system, playing crucial roles in monitoring and modulating neuronal activity (Vidal-Itriago et al., 2022).

Recent studies have revealed that the dura and meningeal compartments may serve as critical channels for communication between the CNS and the immune system. Numerous immune cells reside in the dura and meninges, and the cerebrospinal fluid (CSF) produced by the choroid plexus in the brain can enter the cervical lymph nodes and cranial bone marrow via the dura lymphatic system (Aspelund et al., 2015; Vera Quesada et al., 2023). This evidence suggests that the brain can regulate the immune system through molecules in the CSF (Kisler and Zlokovic, 2022; Mazzitelli et al., 2022; Pulous et al., 2022). Additionally, inflammatory factors released at injury sites can stimulate and regulate peripheral nerves through specific cytokines, such as pathogen-associated molecular patterns, TNF, and IL-1ß (Bourhy et al., 2022). Using optogenetics and chemogenetics, Poller and colleagues demonstrated that brain motor circuits can regulate neutrophil mobilization, distribution, and function during stress through skeletal-muscle-derived chemokines (Poller et al., 2022). At the same time, stress can mobilize bone marrow-derived monocytes to the brain via the hypothalamic-pituitary-adrenal (HPA) axis, exacerbating neuroinflammation and worsening anxiety-like behaviors (Niraula et al., 2018). These findings indicate that although the brain is immune-privileged, it still regulates and communicates with the immune system. Conversely, the immune system also affects brain function. Dysregulation of the immune system is a critical cause and shared characteristic of neurological disorders, such as cerebral ischemia, intracerebral hemorrhage, brain trauma, and neurodegenerative diseases. In brain injury, microglia are activated and participate in neuroinflammatory responses, BBB permeability, the removal of dead cells and debris, and subsequent neurogenesis, synaptogenesis, and myelination through the release of soluble factors and phagocytic capacity (Paolicelli et al., 2022). Meanwhile, BBB destruction promotes mononuclear cell infiltration from the peripheral blood, facilitating crosstalk between the CNS and the immune system (Buckley and McGavern, 2022). As such, neuroimmune communication is not only an essential pathophysiological mechanism but also a potential therapeutic target for brain injury.

Although the neuroinflammatory response to brain injury has both favorable and unfavorable aspects, existing research suggests that nearly all current brain injury treatments can achieve neuroprotection by inhibiting the neuroinflammatory response.

## Neuroprotection against synthetic drugs and natural extracts

Neuroinflammation can exacerbate both acute and secondary injury and lead to adverse long-term outcomes in brain injury. Based on the inflammatory signaling pathway, neuroscientists have found many drugs for the treatment of brain injury in animal studies, such as salvinorin A (a highly selective kappa opioid receptor agonist) (Misilimu et al., 2022), morphine (Rahimi et al., 2021), ACT001 (a sesquiterpene lactone derivative) (Cai et al., 2022), febuxostat (Wang et al., 2022), ethyl pyruvate (Shi et al., 2015), and amantadine (Leclerc et al., 2021). Traditional Chinese medicine formulas and natural extracts have been confirmed to be neuroprotective by regulating the neuroinflammatory response. These include Hu'po Anshen decoction (Shen et al., 2022), cordycepin (Wei et al., 2021), sinomenine (Hong et al., 2022), Rhodiola crenulate (Xie et al., 2022), parthenolide (Ding et al., 2022), and breviscapine (Pengyue et al., 2017). On this topic, Sun et al. found that casein kinase 2 can regulate NR2B phosphorylation, reduce neuroinflammation, and attenuate brain injury induced by intracerebral hemorrhage, and Cui et al. summarized the platelet-Tregs interaction in neuroinflammation after stroke: their works provide some new potential therapeutic targets for the regulation of neuroinflammation and treatment of brain injury. In addition, Wang et al. found that fo cused ultrasounds can improve the delivery of natural extracts into the brain. These studies will promote the development and application of drugs for the treatment of brain injury. Nevertheless, there is still a need for advancement and research in the clinical application of these synthetic drugs (Kakehi and Tompkins, 2021). Although Chinese traditional medicine has been widely used for the treatment of brain injury, it has been administered as an adjunctive treatment only, and its clinical effectiveness needs to be further verified.

## Neuroprotection through rehabilitation

To date, rehabilitation is a widely used and effective treatment for brain injury in the clinic. Rehabilitation programs include stimulation with electricity, magnetism, light, and heat on the CNS and peripheral nervous system (PNS), along with physical therapy, virtual reality, motion imagination, and so on. Although the neuroprotective mechanism of rehabilitation involves multiple targets and multiple factors, inflammatory regulation is its pivotal process. Transcranial-direct-current-stimulation (TDCS) can decrease microglial activation and reduce the release of pro-inflammatory cytokines in experimental stroke models (Zhang et al., 2020; Kaviannejad et al., 2022a,b; Walter et al., 2022). Peripheral nerve electrical stimulation not only promotes nerve regeneration and ameliorates dysfunction but also regulates the neuroinflammatory response in the CNS and PNS (Chu et al., 2022). Exercise immediately after a stroke inhibits the activation of astrocytes and microglia cells and decreases the release of proinflammatory cytokines (Zhang et al., 2017). On this topic, Luo et al. observed that repetitive transcranial magnetic stimulation (TMS) inhibits inflammatory signaling pathways and promotes anti-inflammatory polarization of microglia in ischemic rats.

## Neuroprotection through rehabilitation treatments using traditional Chinese medicine

Traditional Chinese medicine has been widely used in rehabilitation for the prevention and treatment of nervous system disorders. Examples include acupuncture and electric acupuncture, moxibustion, Tai Chi, Baduanjin, Yi Jin Jing, and five-fowl play (Zhang et al., 2017). The neuroprotective mechanisms of acupuncture for brain injury have been relatively well studied. In the traumatic brain injury model, researchers found that acupuncture inhibits M1 polarization of microglia by regulating RhoA/ROCK2 and TLR4/TRIF/MyD88 signaling pathways, thereby mitigating neuroinflammation in the acute, subacute, and chronic phases after TBI (Cavalli et al., 2018; Zhu et al., 2020; Cao et al., 2022). In stroke, electric acupuncture increases miR-223 and α7nAChR protein levels, inhibits NLRP3 inflammasome activation, and alleviates neuroinflammation (Jiang et al., 2019; Sha et al., 2019; Xin et al., 2022).

## Neuroprotection through stem cell-based therapy

Brain injury leads to the loss of neural cells. Stem cells (such as neural stem cells and mesenchymal stem cells), which can proliferate, migrate, and differentiate into neural cells, can take part in nerve repair and neural plasticity by replacing the injured neural cells (Liu et al., 2022). Also, transplanted stem cells can suppress the neuroinflammatory response by sequestering the inflammatory/immune cells at the injury site and releasing anti-inflammatory factors (Mashkouri et al., 2016; Borlongan and Rosi, 2022). In addition, recent evidence indicates that the stem cells modulate microglia M1/M2 phenotypes and suppress NLRP3 inflammasome-mediated inflammation by secreting an exosome rich in multiple miRNAs (Liu et al., 2021). It is worth noting that stem cell-derived exosomes can be genetically reconstructed and deliver functional bioactive molecules (Liu et al., 2021). Thus, stem cell-derived exosomes will promote the clinical application of stem cell-based therapy in the treatment of brain injury because they can cross the blood-brain barrier more easily, cannot block blood vessels, do not induce malignant transformation, and can be genetically reconstructed.

Overall, neuroimmune communication is correlated with the physiological functions of various tissues and organs and the internal environmental balance. Dysregulation of neuroimmune communication homeostasis is both the underlying pathological mechanism and the potential therapeutic target in brain injury. A thorough clarification of the cellular and molecular mechanisms of neuroimmune communication in brain injury is an essential and necessary endeavor in the search for new therapeutic targets for this type of disorder.

## Author contributions

All authors listed have made a substantial, direct, and intellectual contribution to the work and approved it for publication.
